# Supplementary material for: MetaRibo-Seq measures translation in microbiomes
Source: Nat Commun. 2020 Jun 29;11:3268. doi: 10.1038/s41467-020-17081-z (PMC7324362; doi:10.1038/s41467-020-17081-z)
Supplement: Supplementary file 10 — Supplementary Data 7 [file 41467_2020_17081_MOESM10_ESM.zip › File2/Confidence_High_Taxonomy/402347_out.krona.html]

Javascript must be enabled to view this page.

members
magnitude
magnitudeUnassigned
count
unassigned
taxon
rank

402347\_out

64

64
superkingdom
2

1239
64
phylum

class
64
186801

186802
order
64

family
64
186803

genus
64
841

1263104

SRS015579\_contig\_number\_7727SRS019397\_contig\_number\_6217SRS098881\_contig\_number\_17140
species
3

species
60
166486

SRS011084\_contig\_number\_23488SRS013940\_contig\_number\_11498SRS013951\_contig\_number\_38940SRS014287\_contig\_number\_contig-100\_508.120587SRS014613\_contig\_number\_20893SRS014855\_contig\_number\_5769SRS014923\_contig\_number\_45737SRS014948\_contig\_number\_15063SRS015065\_contig\_number\_2606SRS015190\_contig\_number\_13458SRS015431\_contig\_number\_52247SRS015578\_contig\_number\_3542SRS015854\_contig\_number\_18065SRS015890\_contig\_number\_7890SRS016335\_contig\_number\_44755SRS018984\_contig\_number\_10665SRS019068\_contig\_number\_46496SRS021484\_contig\_number\_22379SRS024388\_contig\_number\_11963SRS024435\_contig\_number\_31379SRS024549\_contig\_number\_4829SRS043001\_contig\_number\_contig-100\_120.68797SRS043411\_contig\_number\_14506SRS043701\_contig\_number\_7365SRS050422\_contig\_number\_23886SRS050925\_contig\_number\_31878SRS052027\_contig\_number\_10360SRS053335\_contig\_number\_15282SRS055017\_contig\_number\_35215SRS056273\_contig\_number\_33957SRS056519\_contig\_number\_18486SRS058070\_contig\_number\_25063SRS063040\_contig\_number\_22427SRS074964\_contig\_number\_35SRS075341\_contig\_number\_25784SRS075716\_contig\_number\_4605SRS076929\_contig\_number\_contig-100\_902.152905SRS077454\_contig\_number\_18905SRS077641\_contig\_number\_13166SRS098061\_contig\_number\_10233SRS098514\_contig\_number\_contig-100\_1128.1128SRS098827\_contig\_number\_13167SRS1041130\_contig\_number\_3971SRS104485\_contig\_number\_1547SRS104975\_contig\_number\_18640SRS105153\_contig\_number\_19187SRS1055034\_contig\_number\_11982SRS142503\_contig\_number\_54334SRS142781\_contig\_number\_4178SRS143070\_contig\_number\_17429SRS143722\_contig\_number\_13300SRS144362\_contig\_number\_19210SRS147919\_contig\_number\_20885SRS148091\_contig\_number\_10062SRS148159\_contig\_number\_17002SRS148424\_contig\_number\_22789SRS148721\_contig\_number\_5941SRS149181\_contig\_number\_contig-100\_204.192201SRS149879\_contig\_number\_27359SRS893378\_contig\_number\_18358

1
species

SRS015264\_contig\_number\_contig-100\_51.122776
2049040
